# EXTL3 could serve as a potential biomarker of prognosis and immunotherapy for prostate cancer and its potential mechanisms

**DOI:** 10.1186/s40001-022-00740-w

**Published:** 2022-07-11

**Authors:** Pingan Chang, Shenglan Chen, Xiumei Chang, Jiaxi Zhu, Qingsheng Tang, Limin Ma

**Affiliations:** 1 Nantong University, Nantong, 226001 Jiangsu China; 2 Department of Urology, The Affiliated Dongtai Hospital of Nantong University, No.2 Fukang West Road, Yancheng, 224200 Jiangsu China; 3 Department of Anesthesiology, The Affiliated Dongtai Hospital of Nantong University, Yancheng, 224200 Jiangsu China; 4 Department of Urology, Affiliated Hospital of Nantong University, No. 20 West Temple Road, Nantong, 226001 Jiangsu China

**Keywords:** EXTL3, Biomarker, Immunotherapy, Prostate cancer, Prognosis

## Abstract

**Background:**

Exostosin like glycosyltransferase 3 (EXTL3) had been reported to be associated with immune deficiency and play prognostic roles in various cancers. However, little is known about the associations between EXTL3 and prostate cancer (PCa). Hence, this article was designed to clarify their associations.

**Methods:**

All original data were downloaded from The Cancer Genome Atlas (TCGA) database. Gene set enrichment analysis (GSEA) and CellMiner database was utilized, respectively, to identify EXTL3-related signaling pathways and drugs. We explored the relationships between EXTL3 expression and immunity to further evaluate the involvement of EXTL3 in response to immunotherapies. LncRNA/RBP/EXTL3 mRNA networks were also identified for its potential mechanism.

**Results:**

Compared with normal prostate samples, EXTL3 was poorly expressed in PCa samples not only in mRNA expression levels, but also in protein expression levels, with worse overall survival (*P* < 0.05) and this gene could be an independent prognostic biomarker for PCa (both *P* < 0.05). EXTL3 was revealed to be markedly linked with seven signaling pathways in PCa by GSEA, including calcium, chemokine, ERBB, JAK STAT, MAPK, WNT, oxidative phosphorylation pathways. EXTL3 expression was also revealed to be significantly associated with MSI, immune cells, immune checkpoint molecules, tumor microenvironment and immune cells infiltration. We further predicted immune responses of EXTL3 gene to immunotherapies by TIDE database and the IMvigor210 cohort. A total of six LncRNA/RBP/EXTL3 mRNA networks were eventually identified for its potential mechanisms.

**Conclusions:**

EXTL3 could serve as a potential biomarker of prognosis and immunotherapy for PCa and six LncRNA/RBP/EXTL3 mRNA networks were also identified for its potential mechanisms.

## Introduction

Prostate cancer (PCa), as the most common male malignancy, ranks the first leading cause of newly estimated cases with 248,530 patients and the second leading cause of death with 34,130 cases in men, in the United States, 2021 [1]. PCa is asymptomatic in the early stage and patients usually seek for doctors when symptoms of hematuria or dysuria occur [2]. There are several risk factors commonly known for PCa including family history, age, ethnicity, lifestyle, exposure (occupational or environmental) and so on [3]. Since 1990s, prostate-specific antigen (PSA) was applied into clinical use and early detection rates of PCa had improved significantly [4]. However, several factors were also reported to be able to affect serum PSA levels, containing age, body mass index (BMI), obesity, hypertriglyceridemia, smoking and so on [5]. Currently, various therapeutic methods are available to PCa, including androgen deprivation therapy, radiotherapy, radical prostatectomy, active surveillance, immunotherapy and so on [6]. Therein, androgen deprivation therapy remained the major therapeutic option for PCa [7]. Although these advances in early diagnosis and further treatment of PCa, the 5-year survival rates in distant stages were merely 30.2% and patients were prone to relapse [8, 9]. Hence, there were broad prospects for exploring novel biomarkers to detect the occurrence, progress and relapse of PCa.

Exostosin like glycosyltransferase 3 (EXTL3), also known as RPR; BOTV; REGR; EXTR1; ISDNA; EXTL1L, was located at chromosome 8p21.1 and belonged to the EXT family, encoding glycosyltransferases to biosynthesize heparan sulfate and taking part in the signal transduction of islet-derived proteins, which played roles in stimulating the growth of islet β-cell, gut immune defenses, tissue regeneration and so on [10]. As reported, Volpi et al. shed light on that mutations in EXTL3 could lead to skeletal dysplasia, developmental delay, and severe immune deficiency [11]. Mizuno et al. found that EXTL3 was also able to enhance NF-κB activity mediated by TNF-α [12]. As for the relationships between EXTL3 and tumors, Karibe et al. suggested that EXTL3 promoter methylation could reduce EXTL3 expression and then result in the loss of heparan sulfate in colorectal cancer [13]. Hara et al. shed light on the ubiquitous expression of regenerating gene (REG) Iα receptor EXTL3 in gastric cancer vessel cells and tumor cells [14]. Zhang et al. revealed the potential receptors of EXTL3 for Reg3A in gastrointestinal cancer [15]. Taken together, above-mentioned researches indicated the important roles of EXTL3 in human diseases.

However, little was known about the associations between EXTL3 and PCa. Therefore, we comprehensively analyzed the prognostic roles of EXTL3 in PCa, based on The Cancer Genome Atlas (TCGA), The Human Protein Atlas (HPA) databases. Sangerbox website was utilized to assess the relationships between EXTL3 expression and immunity and Tumor Immune Dysfunction and Exclusion (TIDE) dataset was applied to predict immune responses. We also used starBase v2.0 to identify LncRNA/RNA binding proteins (RBP)/EXTL3 axes for its potential mechanisms. This study offered insights to provide a novel therapeutic target and evidences of EXTL3 in anticancer immunotherapy for PCa.

## Materials and methods

### Data download and EXTL3 gene expression

All original data in this article were downloaded from TCGA (TCGA-PRAD, http://cancergenome.nih.gov/) database, containing 499 PCa tumor tissues and 52 adjacent normal prostate tissues. When TCGA-PRAD samples were short of key clinical information, they shall be excluded. Then, we extracted the EXTL3 single gene matrix and corresponding clinical data for further analyses. TIMER database (https://cistrome.shinyapps.io/timer/) was utilized to reveal the EXTL3 mRNA expression in pan-cancer [16] and HPA database (https://www.proteinatlas.org/) was applied to show the EXTL3 protein expression in PCa tumor tissues and adjacent normal prostate tissues [17].

### R software data processing

All analyses in this article were performed by R software version 4.1.1 (https://www.r-project.org/). The “limma” R package was utilized to display the EXTL3 mRNA distribution in PCa tumor tissues and adjacent normal prostate tissues, with the threshold of adjusted *P*-value (FDR) < 0.05 and |log2-fold change|≥ 1. K–M survival analysis was performed to see the survival differences in high-EXTL3 and low-EXTL3 subgroups, classified by its median expression. A total of 7 clinical factors (Gleason’s score, age, lymph nodes, staged T, cancer status, PSA value, staged N) and EXTL3 expression were enrolled in this article to conduct univariate/multivariate Cox regression analyses to find independent prognostic factors for PCa, with the cutoff criteria of *P* < 0.05 [18]. The “rms” R package was utilized to establish a nomogram based on Gleason’s score, age, lymph nodes, staged T, cancer status, PSA value, staged N, recurrence and EXTL3. C-index, 5-year ROC and calibration curves were carried out to assess its performance [19].

To predict the immune responses of EXTL3 to immunotherapies, TIDE database (http://tide.dfci.harvard.edu/) was utilized to calculate TIDE score, T cell dysfunction score, immune exclusion score in high-EXTL3 and low-EXTL3 subgroups, classified by its median expression. When patients had a higher TIDE score, they shall have a higher chance of immune exclusion, suggesting a less possibility of benefiting from immunotherapy [20]. We also selected the IMvigor210 cohort to predict the immune responses of EXTL3 to anti-PD-L1 treatment atezolizumab in different EXTL3 subgroups [21]. *P* < 0.05 were regarded as statistical significance. CellMiner database (https://discover.nci.nih.gov/cellminer/) was applied to reveal the associations between EXTL3 expression and drug sensitivity IC50 values, with the threshold of correlation coefficient  ≥ 0.3 and *P* < 0.01 [22].

### Gene set enrichment analysis (GSEA) software and Sangerbox tools

GSEA software version 4.0.0 was downloaded from GSEA website (http://www.gsea-msigdb.org/gsea/index.jsp) and gene set of “c2.cp.kegg.v7.1.symbols.gmt” was obtained from Molecular Signatures Database (MSigDB). We utilized GSEA software to identify EXTL3-relevant signaling pathways, according to EXTL3 expression in TCGA-PRAD, with the cutoff criteria of |normalized enrichment score (NES)|> 1.5 as well as nominal *P* < 0.05 [23].

As previously published articles described [24, 25], we utilized the single gene pan-cancer tools in Sangerbox version 2.0 website (http://www.sangerbox.com/tool) to evaluate the relationships between EXTL3 expression based on TCGA-PRAD and immune cells, microsatellite instability (MSI), immune checkpoint molecules, tumor neoantigen burden (TNB), tumor microenvironment, tumor mutational burden (TMB), immune cells infiltration, with the cutoff criteria of *P* < 0.001. STRING database (https://www.string-db.org/) was also searched by us to display EXTL3-involved protein–protein interaction (PPI) networks [26].

### StarBase v2.0 identified EXTL3-related mechanisms

As previously published articles described [27–29], we utilized the StarBase v2.0 website (https://starbase.sysu.edu.cn/) to identify EXTL3-related mechanisms of LncRNA/RBP/mRNA networks. We first searched EXTL3-targeted RBPs with the cutoff criteria of pan-cancer ≥ 10 as well as medium stringency (≥ 2). Then, the selected RBP-targeted LncRNAs were sought once again with the cutoff criteria of pan-cancer ≥ 10, hub LncRNAs in TCGA PRAD (LncRNAs were differently expressed in TCGA-PRAD with threshold of |log2-fold change|≥ 1, FDR < 0.05, and were significantly associated with survival with threshold of *P* < 0.05), LncRNAs positively correlated with EXTL3 in TCGA-PRAD (correlation coefficient ≥ 0.3 and *P* < 0.001), medium stringency (≥ 2). Cytoscape 3.6.1 software was finally utilized by us to display the LncRNA/RBP/EXTL3 mRNA networks.

## Results

### EXTL3 gene expression and survival prognosis in PCa

**Fig. 1:**
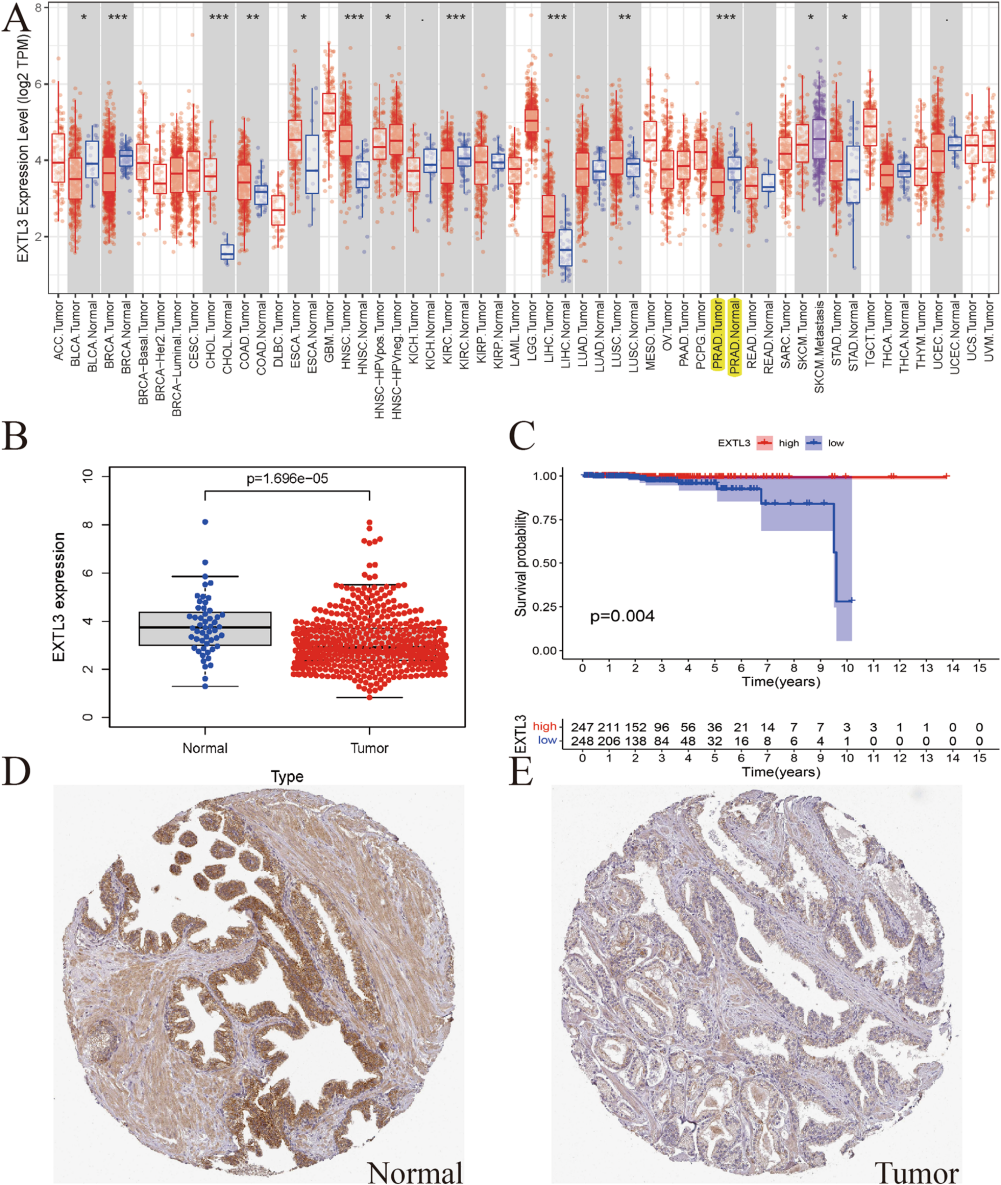
EXTL3 gene expression and survival prognosis in PCa: **A** EXTL3 mRNA expressions in pan-cancer from TCGA; **B** EXTL3 mRNA expressions in PCa from TCGA; **C** survival analysis of EXTL3 in PCa from TCGA; immunohistochemistry of EXTL3 protein expression in **D** normal prostate tissues and **E** PCa tissues from HPA database; *P < 0.05; **P < 0.01;***P < 0.001

Figure 1A details EXTL3 mRNA expressions in pan-caner. It was poorly expressed in cancers including BLCA, BRCA, KIRC, PRAD and highly expressed in cancers containing CHOL, COAD, ESCA, LIHC, LUSC, STAD (all *P* < 0.05). Figure 1B exclusively shows the EXTL3 mRNA expression was poorly expressed in TCGA PCa samples, compared with normal prostate samples (*P* = 1.696e− 05). Survival analysis indicated that patients in high-EXTL3 group shall have a longer overall survival (OS) than patients in low-EXTL3 group (*P* = 0.004; Fig. 1C). Similar to EXTL3 mRNA expression, EXTL3 protein expression was poorly expressed in PCa tissues, compared with normal prostate tissues by CAB025387 antibody in HPA database (Fig. 1D, E). All of these indicated the anticarcinogenic role of EXTL3 in PCa.

### Prognostic values of EXTL3 in PCa from TCGA and nomogram establishment

**Fig. 2:**
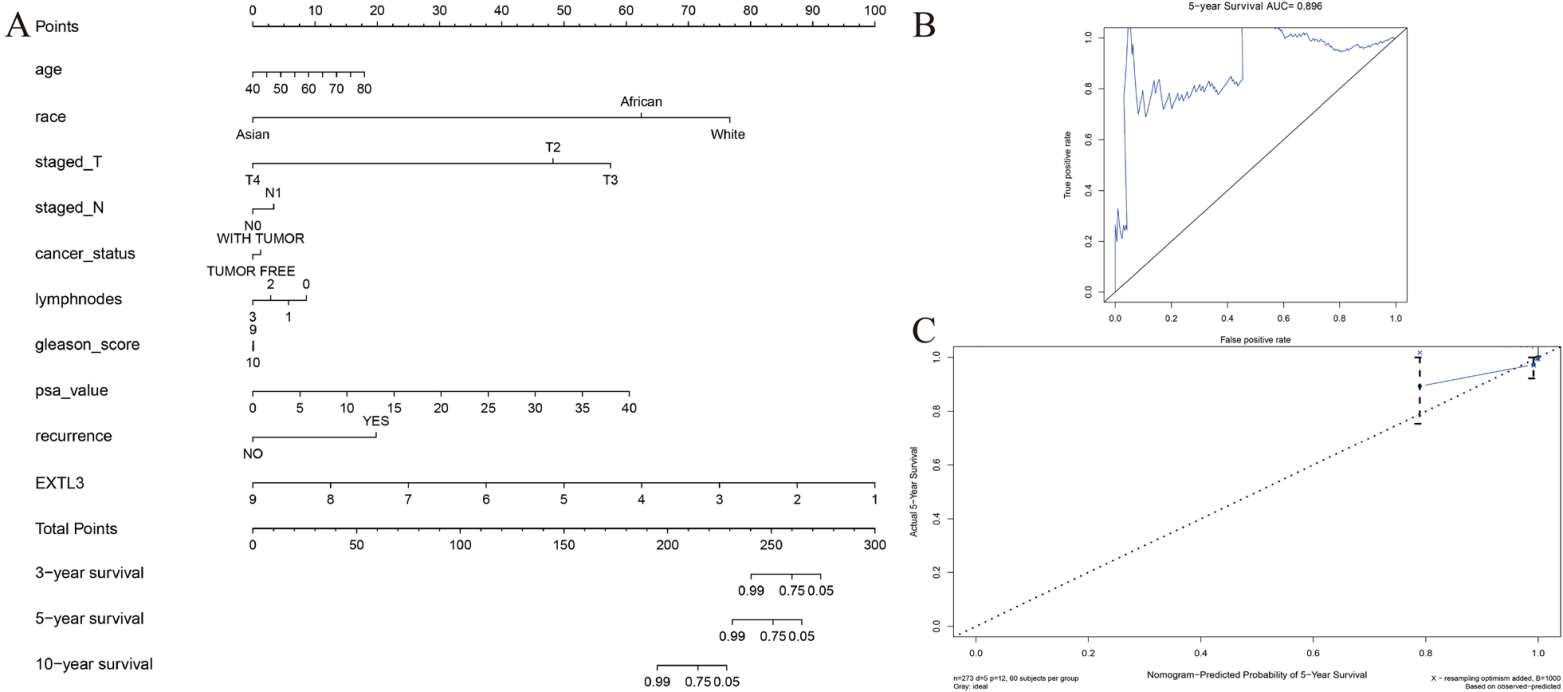
Nomogram establishment for PCa from TCGA: **A** nomogram; **B** 5-year ROC curve; **C** 5-year calibration curve

Univariate Cox regression analysis showed that EXTL3 (HR = 0.201, 95%CI = 0.051–0.789, *P* = 0.021) and PSA value (HR = 1.214, 95%CI = 1.031–1.429, *P* = 0.020) were both prognostic factors in PCa from TCGA. Multivariate Cox regression analysis indicated that EXTL3 (HR = 0.119, 95%CI = 0.022–0.657, *P* = 0.015) was an independent prognostic biomarker for PCa from TCGA (Table 1). Univariate and multivariate Cox regression analyses revealed the independent prognostic ability of EXTL3 in PCa. To further predict PCa patients’ OS possibilities, we established a nomogram based on Gleason’s score, age, lymph nodes, staged T, cancer status, PSA value, staged N, recurrence and EXTL3, with the help of the “rms” R package (Fig. 2A). C-index as well as the area under the curve (AUC) value of 5-year ROC curve of this nomogram were 0.921, 0.896, respectively (Fig. 2B). 5-year calibration curves confirmed the good performance of this nomogram (Fig. 2C).

**Table 1:** Univariate and multivariate Cox analysis based on EXTL3 and clinicopathologic characteristics of OS for PCa

id	Univariate analysis				Multivariate analysis			
	HR	HR.95L	HR.95H	*P *value	HR	HR.95L	HR.95H	*P *value
Age	0.972997	0.855266	1.106936	0.677408	1.006836	0.886295	1.14377	0.916609
Staged_T	2.94079	0.470691	18.3735	0.24855	4.806752	0.117532	196.5834	0.406996
Staged_N	5.648441	0.791027	40.3335	0.084305	2.112223	0.021796	204.6929	0.748647
Cancer_status	5.238172	0.868125	31.60655	0.070957	8.208636	0.511568	131.716	0.137112
Lymphnodes	1.981361	0.925045	4.243892	0.078496	0.666342	0.043656	10.17071	0.770338
Gleason_score	1.31901	0.514518	3.381395	0.564308	0.919313	0.253578	3.332847	0.89813
Psa_value	1.213885	1.031111	1.429057	0.019916	1.28322	0.893096	1.843759	0.17748
EXTL3	0.201004	0.051223	0.788756	0.021439	0.119168	0.021608	0.657197	0.014614

### EXTL3-related signaling pathways in PCa from TCGA

**Fig. 3:**
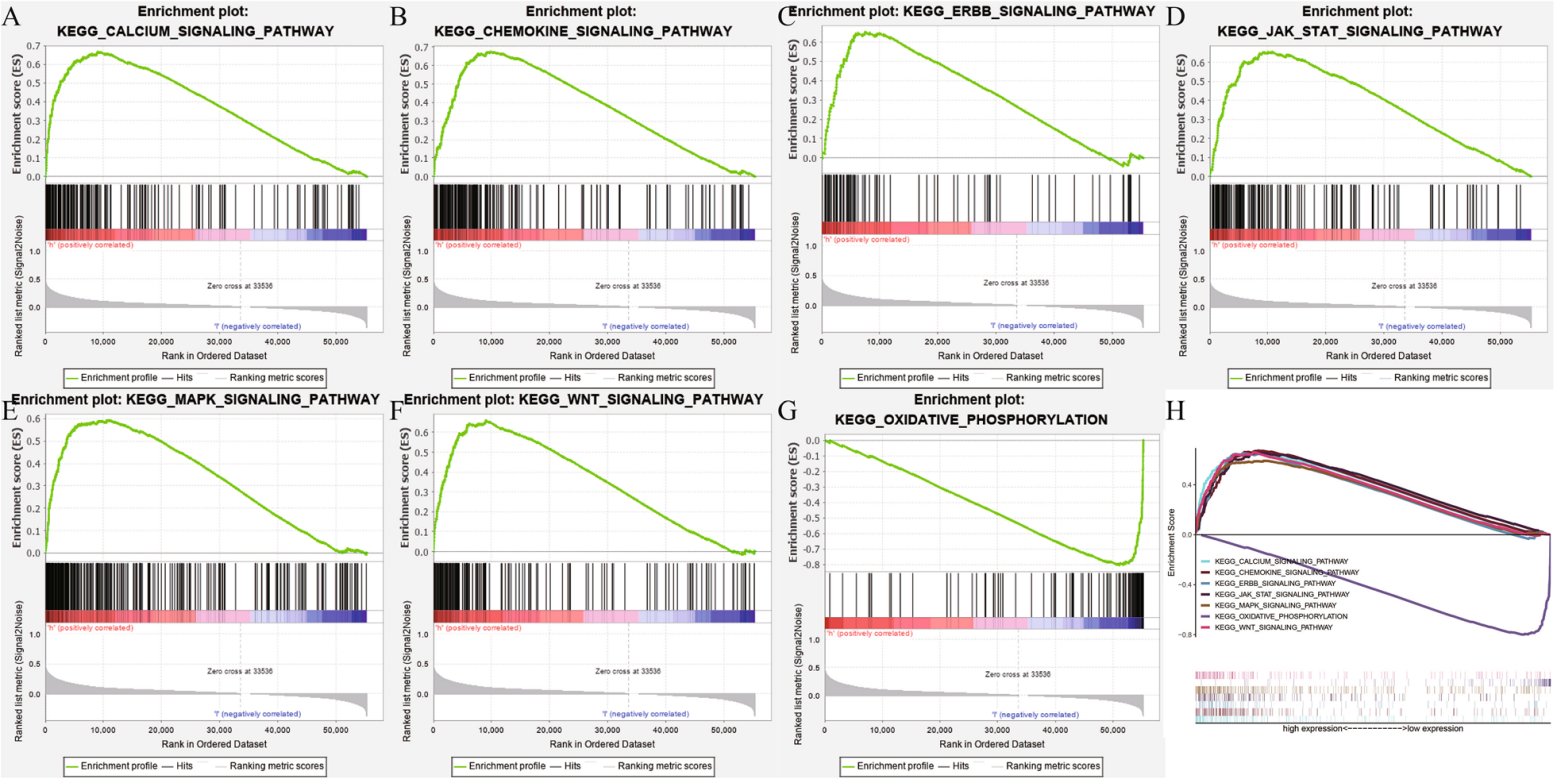
EXTL3-related signaling pathways: **A** calcium pathway; **B** chemokine pathway; **C** ERBB pathway; **D** JAK STAT pathway; **E** MAPK pathway; **F** WNT pathway; **G** oxidative phosphorylation pathway; **H** all of these seven pathways

Based on the gene set of “c2.cp.kegg.v7.1.symbols.gmt” obtained from MSigDB, we utilized the GSEA version 4.0.0 software to identify EXTL3-relevant signaling pathways in PCa. As shown in Fig. 3 and Table 2, EXTL3 was significantly associated with calcium pathway (NES = 2.511; nominal *P* < 0.001), chemokine pathway (NES = 2.284; nominal *P* < 0.001), ERBB pathway (NES = 2.303; nominal *P* < 0.001), JAK STAT pathway (NES = 2.281; nominal *P* < 0.001), MAPK pathway (NES = 2.335; nominal *P* < 0.001), WNT pathway (NES = 2.416; nominal *P* < 0.001), oxidative phosphorylation pathway (NES = − 2.251; nominal *P* < 0.001) in PCa. All of these indicated the potential pathways related to EXTL3 in PCa from TCGA.

**Table 2:** Gene set enrichment analysis results

MSigDB collection	Gene set name	NES	NOM *P*-val	FDR *q*-val
c2.cp.kegg.v7.1 symbols.gmt	KEGG_CALCIUM_SIGNALING_PATHWAY	2.511	< 0.001	< 0.001
	KEGG_CHEMOKINE_SIGNALING_PATHWAY	2.284	< 0.001	< 0.001
	KEGG_ERBB_SIGNALING_PATHWAY	2.303	< 0.001	< 0.001
	KEGG_JAK_STAT_SIGNALING_PATHWAY	2.281	< 0.001	< 0.001
	KEGG_MAPK_SIGNALING_PATHWAY	2.335	< 0.001	< 0.001
	KEGG_WNT_SIGNALING_PATHWAY	2.416	< 0.001	< 0.001
	KEGG_OXIDATIVE_PHOSPHORYLATION	− 2.251	< 0.001	< 0.001

### Correlations between EXTL3 expression and MSI, TNB, TMB, immunity

**Fig. 4:**
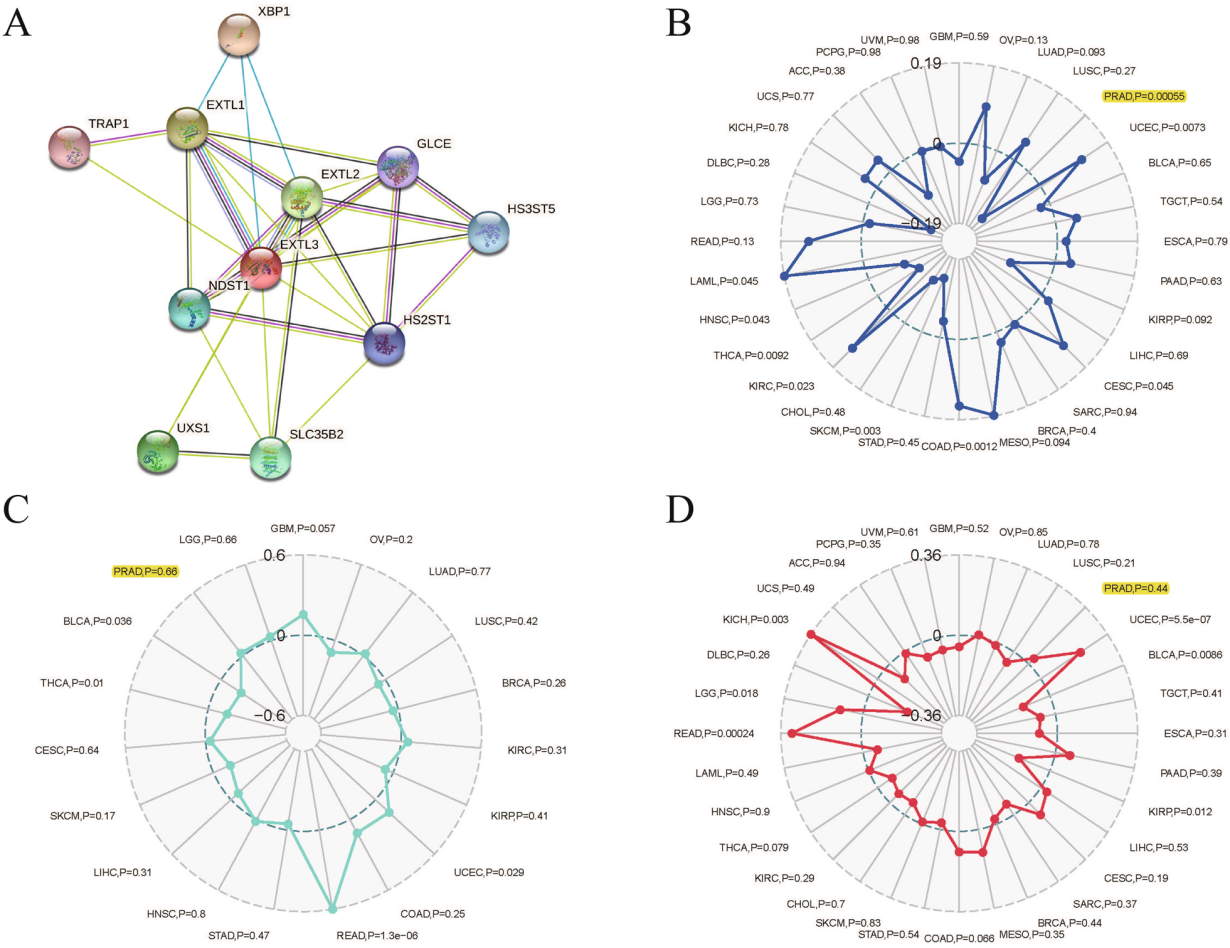
Correlations between EXTL3 expression and **A** PPI networks; **B** MSI; **C** TNB; **D** TMB

We utilized the Sangerbox version 2.0 website single gene pan-cancer tools to evaluate the relationships between EXTL3 expression and immune cells, microsatellite instability (MSI), immune checkpoint molecules, tumor neoantigen burden (TNB), tumor microenvironment, tumor mutational burden (TMB), immune cells infiltration. EXTL3-involved PPI networks are displayed in Fig. 4A. MSI was significantly related to EXTL3 expression (*P* = 0.00055) in TCGA-PRAD (Fig. 4B). TNB (*P* = 0.66) and TMB (*P* = 0.44) did not have significant associations with EXTL3 expression in TCGA-PRAD (Fig. 4C, D).

**Fig. 5:**
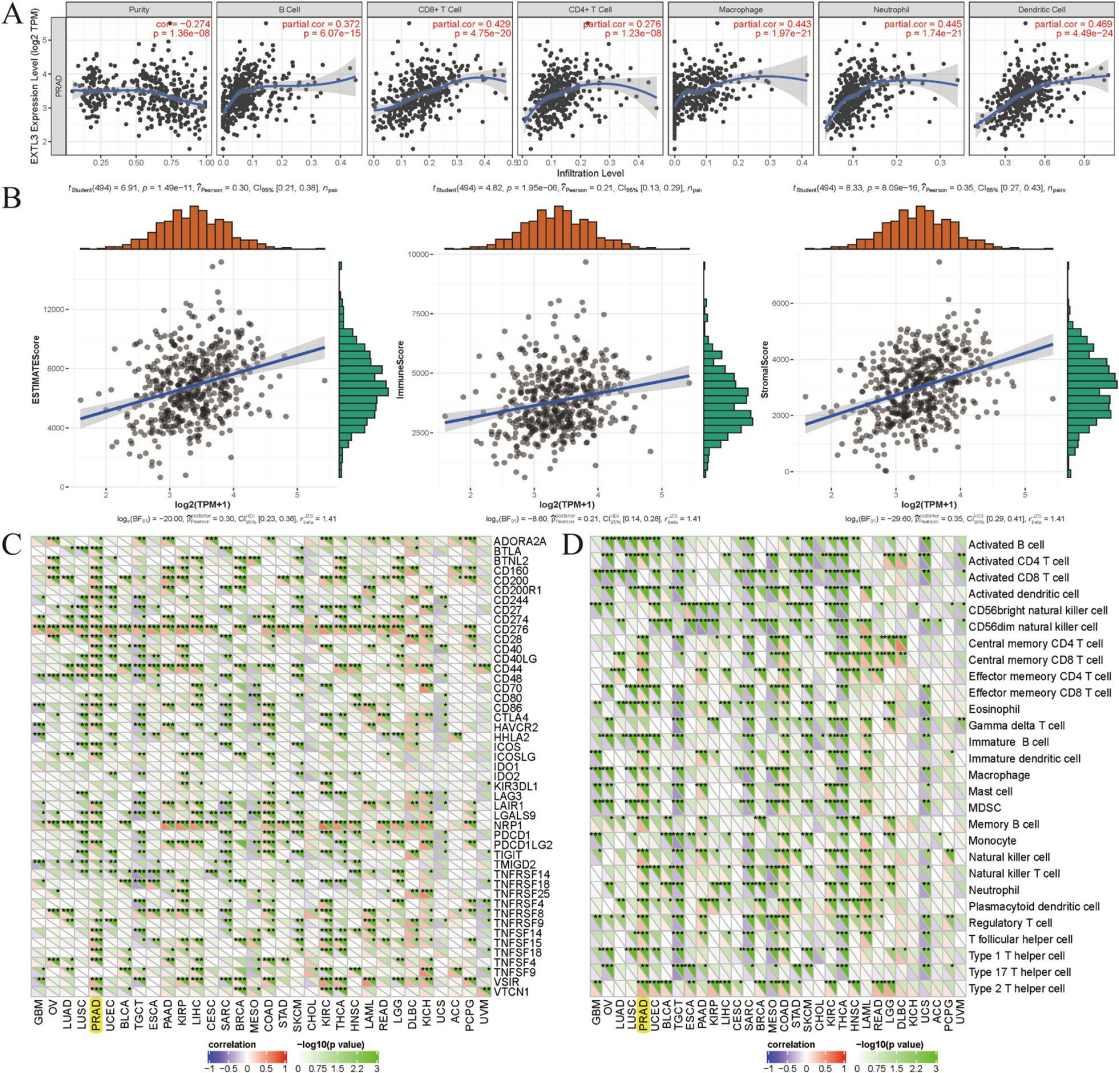
Correlations between EXTL3 expression and **A** immune cells infiltration; **B** tumor microenvironment; **C** immune checkpoint molecules; **D** immune cells; *P < 0.05; **P < 0.01;***P < 0.001

Four aspects of immunity were analyzed, including immune cells, immune checkpoint molecules, tumor microenvironment, immune cells infiltration. Figure 5A presents the correlations between EXTL3 expression and immune cells infiltration levels. We could find that EXTL3 expression was significantly linked with infiltration levels of dendritic cells, B cells, neutrophil cells, CD8+T cells, macrophage cells, CD4+T cells (all *P* < 0.001). As for tumor microenvironment, EXTL3 expression was also remarkably correlated with ESTIMATEScore, ImmuneScore, StromalScore (all *P* < 0.001; Fig. 5B). In terms of the correlations between EXTL3 expression and immune cells, immune checkpoint molecules, EXTL3 expression was markedly involved with CD274, BTLA, CTLA4, CD27, CD244, CD276, activated B cell, Activated CD4 T cell, activated dendritic cell, central memory CD4 T cell, MDSC, type 2 T helper cell and so on (all *P* < 0.05; Fig. 5C, D). All of these indicated significant correlations between EXTL3 expression and immunity.

### The involvement of EXTL3 in response to immunotherapies

**Fig. 6:**
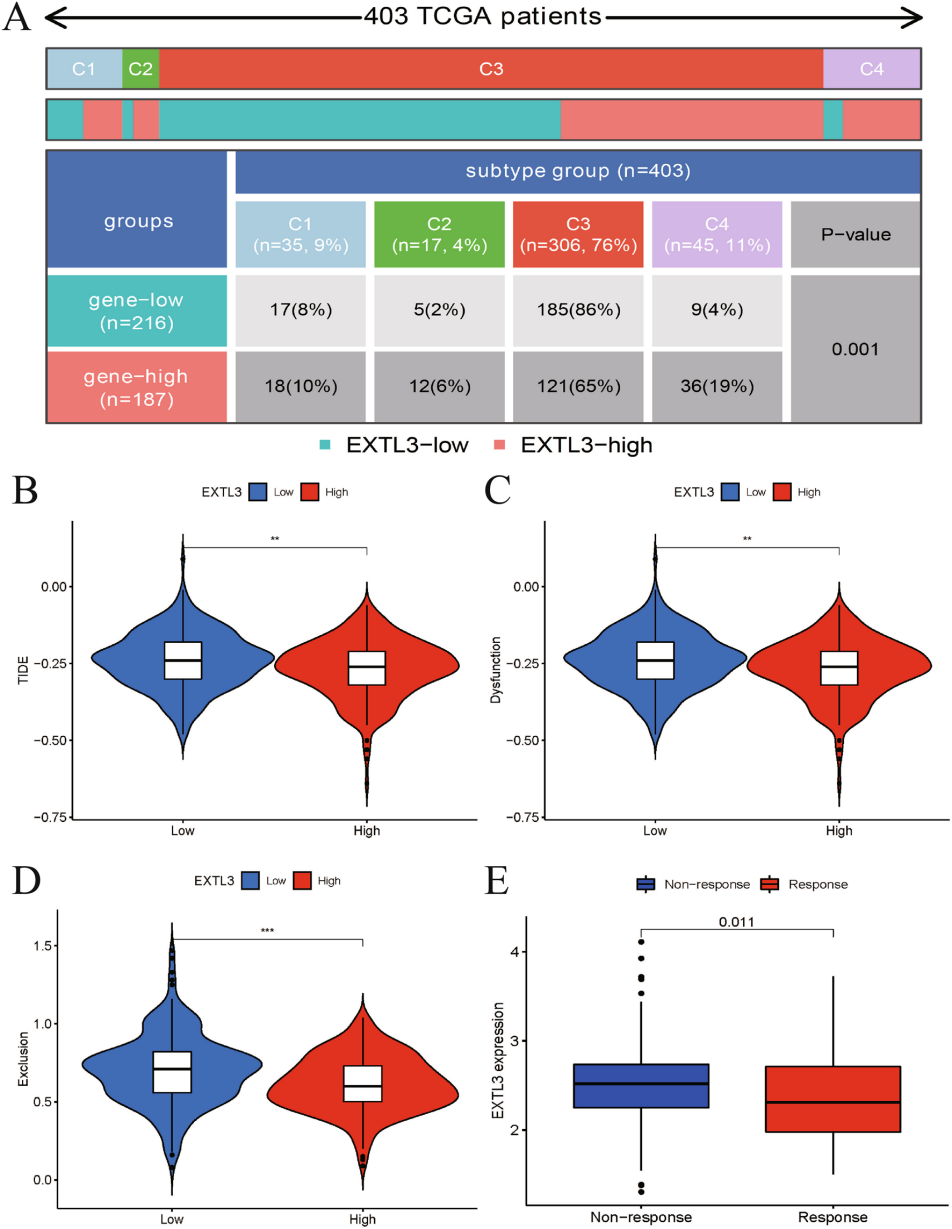
Immune responses of EXTL3 gene to immunotherapies: **A** distribution of EXTL3 in pan-cancer immune subtypes; **B** TIDE score in different EXTL3 subgroups; **C** T cell dysfunction score in different EXTL3 subgroups; **D** immune exclusion score in different EXTL3 subgroups; **E** immune responses of EXTL3 to anti-PD-L1 treatment atezolizumab in the IMvigor210 cohort. **P < 0.01;***P < 0.001

Based on previously published articles and above-mentioned results, EXTL3 had significant associations with immunity. Moreover, the distribution of EXTL3 in pan-cancer immune subtypes is detailed in Fig. 6A (*P *= 0.001). We utilized TIDE database to predict the involvement of EXTL3 in response to immunotherapies by calculating TIDE score, T cell dysfunction score, immune exclusion score in different EXTL3 subgroups. As presented in Fig. 6B–D, low-EXTL3 subgroup was associated with higher TIDE, T cell dysfunction, immune exclusion scores, suggesting a less possibility of benefiting from immunotherapy of low-EXTL3 subgroup patients. We also selected the IMvigor210 cohort to predict the immune responses of EXTL3 to anti-PD-L1 treatment atezolizumab in different EXTL3 subgroups. Figure 6E indicates that patients in high-EXTL3 subgroup were more sensitive to anti-PD-L1 treatment atezolizumab.

### ***EXTL3-***related drugs from CellMiner database

**Fig. 7:**
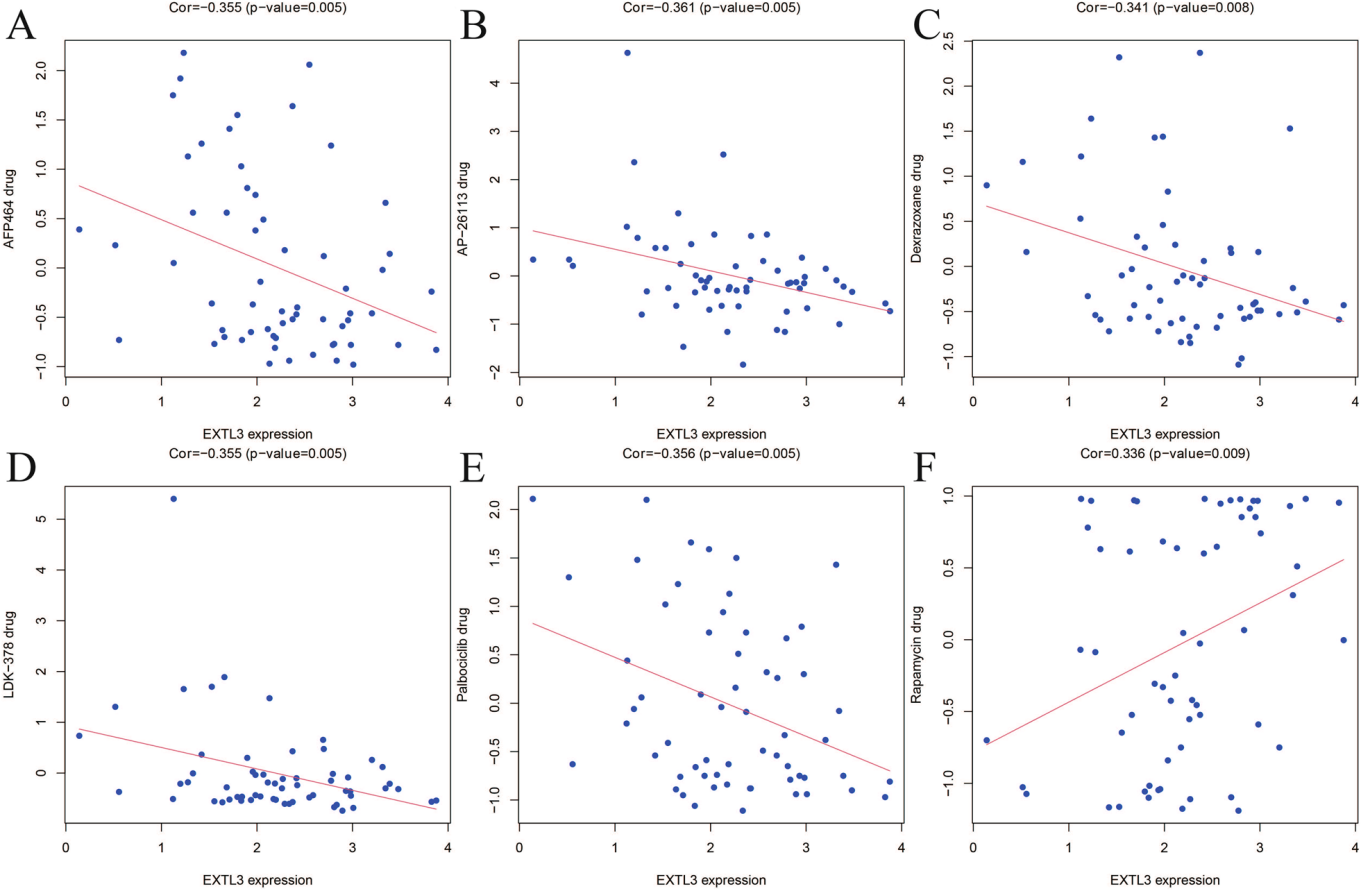
Associations between EXTL3 expression and **A** AFP464; **B** AP-26113; **C** Dexrazoxane; **D** LDK-378; **E** palbociclib; **F** rapamycin; in the CellMiner database

We applied CellMiner database to reveal the associations between EXTL3 expression and drug sensitivity IC50 values, with the threshold of correlation coefficient ≥ 0.3 and *P* < 0.01. As detailed in Fig. 7, EXTL3 expression was dramatically correlated with AFP464 (correlation coefficient = − 0.355; *P* = 0.005), AP-26113 (correlation coefficient = − 0.361; *P *= 0.005), dexrazoxane (correlation coefficient = − 0.341; *P *= 0.008), LDK-378 (correlation coefficient = − 0.355; *P* = 0.005), palbociclib (correlation coefficient = − 0.356; *P* = 0.005), rapamycin (correlation coefficient = 0.336; *P* = 0.009). All of these indicated that five negatively correlated drugs (AFP464, AP-26113, dexrazoxane, LDK-378, palbociclib) had the potential to prevent the progress of PCa, while rapamycin might promote this disease.

### EXTL3-related mechanism of LncRNA/RBP/EXTL3 mRNA networks

**Fig. 8:**
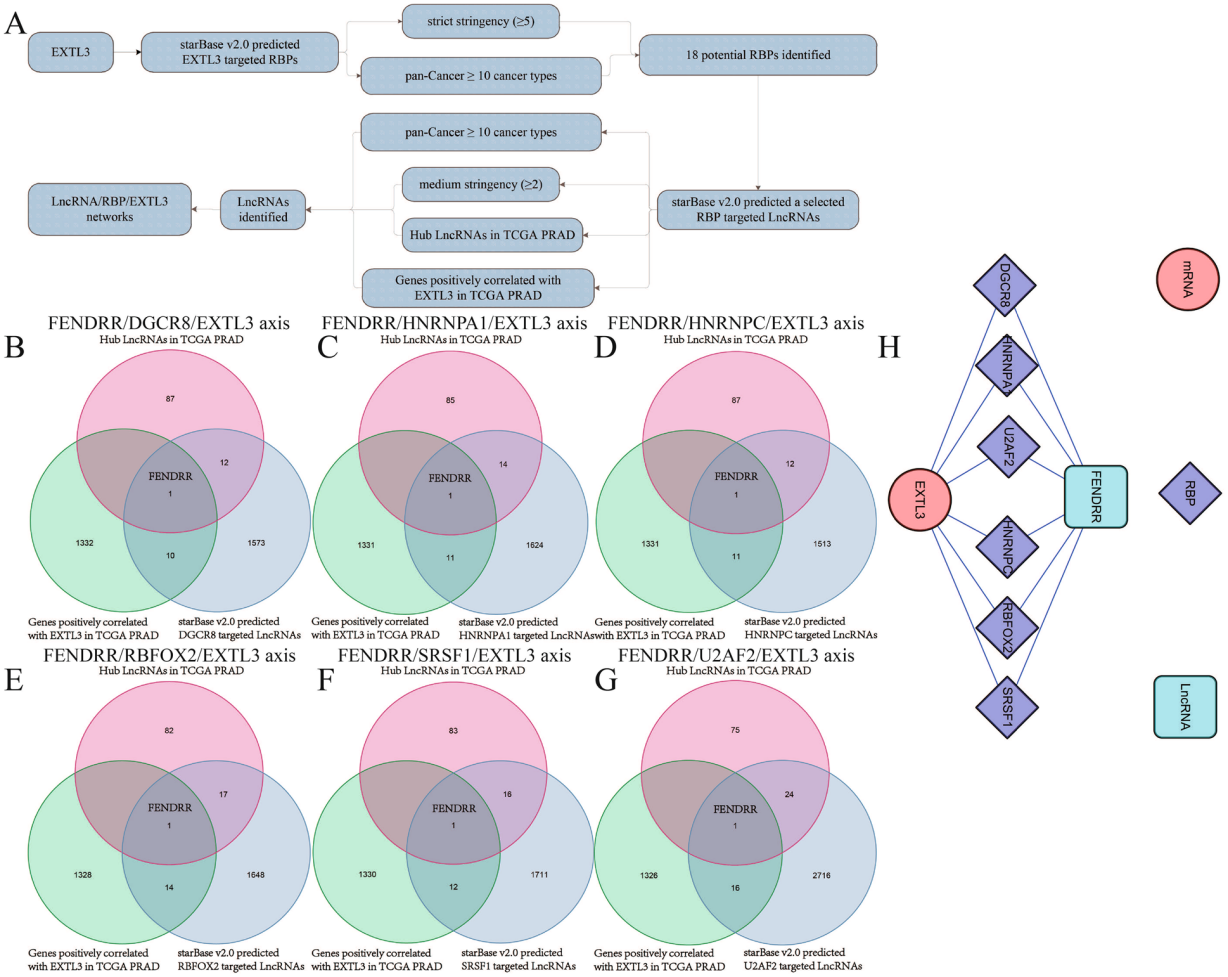
EXTL3-related mechanism of LncRNA/RBP/EXTL3 mRNA networks: **A** flowchart; **B** Venn diagrams of FENDRR/DGCR8/EXTL3 axis; **C** Venn diagrams of FENDRR/HNRNPA1/EXTL3 axis; **D** Venn diagrams of FENDRR/HNRNPC/EXTL3 axis; **E** Venn diagrams of FENDRR/RBFOX2/EXTL3 axis; **F** Venn diagrams of FENDRR/SRSF1/EXTL3 axis; **G** Venn diagrams of FENDRR/U2AF2/EXTL3 axis; **H** LncRNA/RBP/EXTL3 mRNA networks by Cytoscape 3.6.1

We utilized the StarBase v2.0 website to identify EXTL3-related mechanisms of LncRNA/RBP/EXTL3 mRNA networks. The detailed flowchart is summarized in Fig. 8A. A total of 18 potential RBPs were identified after first searching RBP-targeted LncRNAs. However, only six LncRNA/RBP/EXTL3 mRNA networks (FENDRR/DGCR8/EXTL3 axis, FENDRR/HNRNPA1/EXTL3 axis, FENDRR/HNRNPC/EXTL3 axis, FENDRR/RBFOX2/EXTL3 axis, FENDRR/SRSF1/EXTL3 axis, FENDRR/U2AF2/EXTL3 axis) were remained after second searching a selected RBP-targeted LncRNAs, based on the results of Venn diagrams (Fig. 8B–G). Cytoscape 3.6.1 software was finally utilized by us to display these six LncRNA/RBP/EXTL3 mRNA networks (Fig. 8H). A total of six RBP-involved mechanisms of LncRNA/RBP/EXTL3 mRNA networks were eventually identified for EXTL3-related mechanisms of PCa in this article.

## Discussion

PCa is an indolent cancer, characterized by its insidious onset, low drug sensitivity, high possibilities of metastasis or relapse, associated with a 5-year survival of less than 30% [30]. As we know, PCa patients’ morbidity and mortality in the United States and Europe were ranked first and second in the male population [31]. In developing countries, its incidence is also increasing year by year [32]. Moreover, as we had described previously, EXTL3 belonged to the EXT family, playing vital roles in predicting various cancers’ prognosis and immune deficiency [13, 14, 33]. However, associations between EXTL3 and PCa had seldom been explored. Hence, in this article, we comprehensively analyzed the prognostic roles of EXTL3 in PCa and assessed the relationships between EXTL3 expression and immunity to predict immune responses. We also identified LncRNA/RBP/EXTL3 axes for its potential mechanisms. Our findings were expected to provide a novel treatment target and evidence of EXTL3 in anticancer immunotherapy for PCa.

Our results shed light on that EXTL3 was poorly expressed in PCa samples not only in mRNA expression levels, but also in protein expression levels, compared with normal prostate samples. Moreover, PCa patients in high-EXTL3 groups shall have a longer OS than patients in poorly expressed groups and this gene could be an independent prognostic biomarker for PCa. Consistent with previously published articles, Zhang et al. revealed the potential receptors of EXTL3 for Reg3A in gastrointestinal cancer [15]. EXTL3 promoter methylation could reduce EXTL3 expression, resulting in the loss of heparan sulfate in colorectal cancer [13] and the ubiquitous expression of regenerating gene (REG) Iα receptor EXTL3 was found in gastric cancer vessel cells and tumor cells [14]. All of these studies indicated the prognostic value of EXTL3 in cancer.

As for TCGA data mining, nomogram was often utilized to intuitively display the relationships between clinical factors and patients’ survival [34, 35]. To further predict PCa patients’ OS possibilities, we established a nomogram in this article based on Gleason’s score, age, lymph nodes, staged T, cancer status, PSA value, staged N, recurrence and EXTL3, with good performance. For single gene, GSEA was commonly applied by us to underline the potential signaling pathways related to this gene [36, 37]. According to our research, GSEA also identified several EXTL3-relevant signaling pathways, including the calcium pathway, chemokine pathway, ERBB pathway, JAK STAT pathway, MAPK pathway, WNT pathway, oxidative phosphorylation pathway. As reported by Tam et al., the IL-6R/JAK/STAT3 signaling pathway activation could lead to the progress of hormone-refractory PCa [38]. Wang et al. reported that GADD45B could promote PCa’s chemosensitivity and cell apoptosis by MAPK signaling pathway [39]. Zhao et al. concluded that AGAP2-AS1/miR-628-5p/FOXP2 axis was able to facilitate the growth of PCa cells by means of WNT signaling pathway [40]. As for oxidative phosphorylation pathways, Xiao et al. revealed that phenethyl isothiocyanate might trigger reactive oxygen species (ROS)-mediated of PCa cells’ death via inhibiting oxidative phosphorylation [41].

MSI, TNB and TMB had been regarded as vital factors influencing cancer patients’ prognosis and they could also be as characterized as biomarkers of immunotherapies [42–44]. Based on our results, MSI was significantly related to EXTL3 expression in TCGA-PRAD, while TMB and TNB did not have significant associations with EXTL3 expression. To further seek EXTL3-related drugs in PCa, CellMiner database was utilized and this database was designed for facilitating the study and selection of anticancer drugs [45, 46]. Our results showed that EXTL3 expression was negatively correlated with AFP464, AP-26113, dexrazoxane, LDK-378, palbociclib, having the potential to prevent the progress of PCa. Moreover, EXTL3 expression was positively correlated with rapamycin, having the potential to promote this disease.

Currently, there had been various types of immunotherapies applied in the treatment of cancer, including cancer vaccines, oncolytic virus therapies, adoptive cell transfer, immune checkpoint inhibitors, cytokine therapies, and so on [47]. Despite the successful application of these immunotherapies, merely a part of patients with cancer could benefit from them [48]. Hence, there was also an urgent need to better understand tumor microenvironment. As reported, the interactions among tumor and immune cells in tumor microenvironment shall result in tumorigenesis, metastasis and relapse [49]. Moreover, immune cell infiltration, as an important part of tumor microenvironment, was significantly linked with tumor progression and could predict immune responses [50]. In our article, four aspects of immunity were analyzed, including immune cells, immune checkpoint molecules, tumor microenvironment, immune cells infiltration levels. As for immune cells infiltration levels, we could find that EXTL3 expression was significantly linked with infiltration levels of dendritic cells, B cells, neutrophil cells, CD8 + T cells, macrophage cells, CD4 + T cells. In terms of tumor microenvironment, EXTL3 expression was also remarkably correlated with ESTIMATEScore, ImmuneScore, StromalScore. For the correlations between EXTL3 expression and immune cells, immune checkpoint molecules, EXTL3 expression was markedly involved with CD274, BTLA, CTLA4, CD27, CD244,CD276, activated B cell, activated CD4 T cell, activated dendritic cell, central memory CD4 T cell, MDSC, Type 2 T helper cell and so on. All of these indicated significant associations between EXTL3 with immunity in PCa.

Although significant responses to immunotherapies had been showed in other metastatic cancers including melanoma, renal cancer or lung cancer, only a small number of PCa sufferers showed responses to immunotherapies, due to complex tumor environment interactions among immune and malignant PCa cells. Currently, immunotherapies for PCa included immune checkpoint inhibitors and vaccine-based therapies [51]. As reported, the only approved PCa immunotherapy was sipuleucel-T vaccine therapy. Moreover, Ipilimumab or olaparib also showed survival benefits in PCa [52]. All of these indicated the urgent need to identify biomarkers for PCa immunotherapy. In this article, we further predicted immune responses of EXTL3 gene to immunotherapies, by means of TIDE database and the IMvigor210 cohort [53, 54]. Based on our results, the low-EXTL3 subgroup was associated with higher TIDE, T cell dysfunction, immune exclusion scores, suggesting a lesser possibility of these patients benefiting from immunotherapy. We also selected the IMvigor210 cohort to predict the immune responses of EXTL3 to anti-PD-L1 treatment atezolizumab in different EXTL3 subgroups, indicating that patients in high-EXTL3 subgroup were more sensitive to anti-PD-L1 treatment atezolizumab. All in all, patients with high EXTL3 expression were prone to benefit from immunotherapy.

Based on above-mentioned, we could easily find that EXTL3 served as an anti-oncogene in PCa. In order to further reveal EXTL3-related mechanisms, we referred to previously published articles as well as the functions of LncRNAs or RBPs [55–57]. Finally, the RBP-involved mechanisms of LncRNA/RBP/mRNA networks were revealed. As previously reported, Zou et al. found that the LINC00324/HuR (RBP)/FAM83B axis could promote the gastric cancer cells’ proliferation [28]. Yu et al. revealed that circ_0003258 could drive prostate cancer metastasis via both a competing endogenous RNA (ceRNA) mechanism of circ_0003258/miR-653-5p/ARHGAP5 and a RBP-involved mechanism of circ_0003258/IGF2BP3 (RBP)/HDAC4 [58]. In this article, the RBP-involved mechanisms of LncRNA/RBP/EXTL3 mRNA networks were identified by us with the help of StarBase v2.0 website. Therein, a total of six LncRNA/RBP/EXTL3 mRNA networks containing the FENDRR/DGCR8/EXTL3 axis, the FENDRR/HNRNPA1/EXTL3 axis, the FENDRR/HNRNPC/EXTL3 axis, the FENDRR/RBFOX2/EXTL3 axis, the FENDRR/SRSF1/EXTL3 axis, the FENDRR/U2AF2/EXTL3 axis, were eventually identified in this article for EXTL3-related mechanisms for PCa.

Several limitations should not be ignored too. Firstly, all analyses were based on bioinformatics analysis, without experimental verification. Further in vivo or in vitro experiments were required to validate the expression of EXTL3, EXTL3-related signaling pathways and its related mechanisms of LncRNA/RBP/EXTL3 mRNA networks. Secondly, most of these analyses were based on EXTL3 mRNA expressions. This might not be consistent with its protein expressions. Thirdly, other information (such as prostate cancer family history, immunotherapy, and so on) was limited in TCGA database. Hence, we currently had difficulties in analyzing these factors. Finally, no targeted EXTL3 therapy was currently available. Hence, we could not directly obtain clinical information and immune responses of this targeted therapy, affecting our results to some certain extent.

## Conclusions

In summary, EXTL3 could serve as an anti-oncogene in PCa and it was found to be markedly linked with seven signaling pathways in PCa by GSEA, including calcium, chemokine, ERBB, JAK STAT, MAPK, WNT, oxidative phosphorylation pathways. EXTL3 expression was also revealed to be significantly associated with MSI, immune cells, immune checkpoint molecules, tumor microenvironment and immune cells infiltration. We further predicted the involvement of EXTL3 in response to immunotherapies by TIDE database and the IMvigor210 cohort. A total of six LncRNA/RBP/EXTL3 mRNA networks including the FENDRR/DGCR8/EXTL3 axis, the FENDRR/HNRNPA1/EXTL3 axis, the FENDRR/HNRNPC/EXTL3 axis, the FENDRR/RBFOX2/EXTL3 axis, the FENDRR/SRSF1/EXTL3 axis, the FENDRR/U2AF2/EXTL3 axis, were eventually identified in this article for its potential mechanisms.

## Data Availability

The RNA-sequencing data and corresponding clinical information were downloaded from the Cancer Genome Atlas (TCGA) database (https://portal.gdc.cancer.gov/).

## Abbreviations

EXTL3: Exostosin like glycosyltransferase 3
PCa: Prostate cancer
TCGA: The Cancer Genome Atlas
GSEA: Gene set enrichment analysis
OS: Overall survival
BMI: Body mass index
REG: Regenerating gene
HPA: The Human Protein Atlas
TIDE: Tumor immune dysfunction and exclusion
RBP: RNA binding proteins
FDR: Adjusted *P*-value
MSigDB: Molecular signatures database
NES: Normalized enrichment score
TNB: Tumor neoantigen burden
TMB: Tumor mutational burden
MSI: Microsatellite instability
PPI: Protein–protein interaction
BLCA: Bladder urothelial carcinoma
BRCA: Breast invasive carcinoma
CHOL: Cholangiocarcinoma
COAD: Colon adenocarcinoma
ESCA: Esophageal carcinoma
HNSC: Head and neck squamous cell carcinoma
KIRC: Kidney renal clear cell carcinoma
LIHC: Liver hepatocellular carcinoma
LUSC: Lung squamous cell carcinoma
PRAD: Prostate adenocarcinoma
SKCM: Skin cutaneous melanoma
STAD: Stomach adenocarcinoma
